# Impact of detector selections on inter‐institutional variability of flattening filter‐free beam data for TrueBeam™ linear accelerators

**DOI:** 10.1002/acm2.12766

**Published:** 2019-11-18

**Authors:** Yoshihiro Tanaka, Yuichi Akino, Hirokazu Mizuno, Masaru Isono, Norihisa Masai, Toshijiro Yamamoto

**Affiliations:** ^1^ Department of Radiation Therapy Japanese Red Cross Society Kyoto Daiichi Hospital Kyoto Japan; ^2^ Oncology Center Osaka University Hospital Suita Osaka Japan; ^3^ Department of Medical Physics and Engineering Osaka University Graduate School of Medicine Suita Osaka Japan; ^4^ Department of Radiation Oncology Osaka International Cancer Institute Osaka Japan; ^5^ Miyakojima IGRT Clinic Osaka Japan; ^6^ Department of Radiation Therapy SaiseikaiNoe Hospital Osaka Japan

**Keywords:** beam data commissioning, detector selection, flattening filter‐free beam, TrueBeam

## Abstract

This study evaluates the type of detector influencing the inter‐institutional variability in flattening filter‐free (FFF) beam‐specific parameters for TrueBeam™ linear accelerators (Varian Medical Systems,Palo Alto, CA, USA). Twenty‐four beam data sets, including the percent depth dose (PDD), off‐center ratio (OCR), and output factor (OPF) for modeling within the Eclipse (Varian Medical Systems) treatment planning system, were collected from 19 institutions. Although many institutions collected the data using CC13 (IBA Dosimetry, Schwarzenbruck, Germany) or PTW31010 semiflex (PTW Freiburg, Freiburg, Germany) ionization chambers, some institutions used diode detectors, diamond detectors, and ionization chambers with smaller cavities. The OCR data included penumbra width, full width at half maximum (FWHM), and FFF beam‐specific parameters, including unflatness and slope. The data measured by CC13/PTW31010 ionization chambers were compared with those measured by all other detectors. PDD data demonstrated the variations within ±1% at the dose fall‐off region deeper than peak depth. The penumbra widths of the OCR measured with the CC13/PTW31010 detectors were significantly larger than those measured with all other detectors (*P* < 0.05). Especially the EDGE detector (Sun Nuclear Corp., Melbourne, FL, USA) and the microDiamond detectors (model 60019; PTW Freiburg) demonstrated much smaller penumbra values compared to those of the CC13/PTW31010 detectors for the 30 × 30 mm^2^ field. There was no difference in the FWHM, unflatness, and slope parameters between the values for the CC13/PTW31010 detectors and all other detectors. OPF curves demonstrated small variations, and the relative difference from the mean value of each data point was almost within 1% for all field sizes. Although the penumbra region exhibited detector‐dependent variations, all other parameters showed tiny interunit variations regardless of the detector type.

## 1 INTRODUCTION

Stereotactic radiotherapy has demonstrated excellent local control of intracranial and extracranial localized tumors.^1^, ^2^, ^3^ Recently, linear accelerators (linacs) equipped with flattening filter‐free (FFF) beams have become commonly used around the world. For conventional treatments, photon beams with high intensities at the center of the beam become flattened by a flattening filter. For stereotactic treatments with small fields, however, a flattened beam is not essential. By eliminating the filter, the beams can deliver a very high dose rate, which decreases the treatment time.^4^, ^5^, ^6^

It has been indicated in several studies that the interunit variability in modern linacs is small, likely owing to the improved manufacturing.^7^ For TrueBeam™ (Varian Medical Systems, Palo Alto, CA, USA) linacs, the vendor‐provided representative beam data (RBD)^8^ are often used for beam modeling within the Eclipse (Varian Medical Systems) treatment planning system (TPS). The RBD were generated on the basis of the beam data of three TrueBeam™ units measured at one institution using a CC13 ionization chamber (IBA Dosimetry, Schwarzenbruck, Germany). Tanaka et al. recently collected 21 sets of TrueBeam™ beam data measured with the CC13 or PTW31010 (PTW Freiburg, Freiburg, Germany) from multiple institutions, and they reported that the interunit variability was very small.^9^ While these ionization chambers are often used for beam data collection, various other detectors with small sensitive volumes, such as ionization chambers with smaller cavities, diode detectors, diamond detectors, and plastic scintillators, are also employed, especially for measuring small‐field beams. It has been demonstrated in many studies that the detector type has an influence on the penumbra of off‐center ratio (OCR) profiles and output factors (OPFs).^10^, ^11^, ^12^, ^13^ Akino et al. previously collected the beam data of Novalis Tx™ (Varian Medical Systems and Brainlab, Munich, Germany) from multiple institutions and reported a detector‐dependent variability.^14^

It has been shown in many studies that the characteristics of FFF beams differ from those of flattened beams in terms of the cone‐shaped OCR, lower effective beam energy affecting the percent depth dose (PDD), photon energy spectrum affecting the water–air stopping power ratio,^15^, ^16^, ^17^ and high dose per pulse affecting the ion recombination coefficient.^18^, ^19^ Moreover, the type of the detector may affect the collected beam data. In addition, there are some dosimetric parameters specialized for FFF beams because of their unique profile shape.^20^ However, no one has reported how detector selection affects these parameters. Here we investigate the impact of detector selection on the FFF beam‐specific parameters for beam data collected from multiple institutions.

## 2 MATERIALS AND METHODS

### 2.1 Data collection

According to institutional agreement, 24 sets of TrueBeam™ data were collected from 19 institutions. All data were measured for modeling within the Eclipse TPS, and treatment fields were collimated with jaws. Data were submitted in the format of the three‐dimensional scanning water phantoms or W2CAD format, a format for data registration of the Eclipse TPS. The field sizes (FSs) of the collected data were 30, 100, and 200 mm square fields. PDD and crossline OCR data were measured with a source‐to‐surface distance of 100 cm. The OPF data were collected in a Microsoft Excel (Microsoft Corp., Redmond, WA, USA) spreadsheet. All collected data were imported in Akilles RT software (RADLabInc., Osaka, Japan) to create a database. The details of the detectors evaluated in this study are listed in Table S1. The number of detectors used for data collection is listed in Tables 1 and 2. Most of the institutions used either the CC13 or PTW31010 semiflex ionization chamber. Only one institution used the PTW30013 Farmer‐type ionization chamber to measure OPF and compared their data with the RBD.

**Table 1:** Number of institutions using each detector and FS for measurements of PDD and OCR

Detector	FS	PDD			OCR		
		30 × 30	100 × 100	200 × 200	30 × 30	100 × 100	200 × 200
PTW60019		1	—	—	2^a^	1	1
EDGE		2	—	—	3	1	1
PTW LA48		—	—	—	1	1	1
CC01/PTW31016		4	2	2	3	1	—
CC04		1	3	3	2	4^a^	2
CC13/PTW31010		11^a^	15^a^	14^a^	6	12	14^a^

Abbreviations: FFF, flattening filter‐free; FS, field size (mm^2^); OCR, off‐center ratio; PDD, percent depth dose.

a Values for 6 MV FFF beams. For 10 MV FFF beams, the number of institutions decreased by one.

**Table 2:** Number of institutions using each detector and FS for measurements of the OPF

Detector	FS (mm^2^)						
	30 × 30	40 × 40	50 × 50	150 × 150	200 × 200	300 × 300	400 × 400
EDGE	1	—	—	—	—	—	—
CC01/PTW pinpoint^a^	3	1	1	—	—	—	—
CC04	2	2	2	2	2	2	2
CC13/PTW31010	12	14	8	13	14	16	14
PTW30013	—	—	—	1	—	1	—

Abbreviations: FS, field size; OPF, output factor.

a PTW PinPoint includes model 31014 and model 31016 ionization chambers.

### 2.2 Data analysis

All PDD data were resampled to the data with a 1 mm interval and normalized at 100 mm depth, as data normalized at the peak depth will be affected by noise around the peak. For each data point, the mean value and standard deviation (SD) of 24 machines were calculated. In order to evaluate the variability, the maximum SD (SD_max_) was calculated for the dose fall‐off region deeper than the peak depth.

For OCR data, the data measured at $d_{\max}$ and a depth of 10 cm were analyzed. Mean (range) values of $d_{\max}$ were 13 mm (12–15 mm) and 23 mm (21–25 mm) for 6 and 10 MV FFF beam, respectively. All data were resampled to the data with a 1 mm interval and normalized using renormalization factors provided by Fogliata et al.^20^ The penumbra width was evaluated for the 20–80% profile region. The full width at half maximum (FWHM) was calculated for 50% of the profiles normalized using the renormalization factors. Unflatness and slope, which are FFF beam‐specific parameters that were proposed by Fogliata et al.,^20^ were calculated. Unflatness was calculated as

(1) $$\text{Unflatness}=\frac{{\text{Dose}}_{\text{central}\;-\;\text{axis}}}{{\text{Dose}}_{\text{X - off - axis}}},$$

where ${\text{Dose}}_{\text{central}\;-\;\text{axis}}$ and ${\text{Dose}}_{\text{X - off - axis}}$ represent the central‐axis dose level and the dose level at a certain off‐axis position, respectively. Slope was calculated as

(2) $$\text{Slope}=\frac{\left(x_{1}-x_{2}\right)\times \left(y_{1}-y_{2}\right)}{{\left(x_{1}-x_{2}\right)}^{2}},$$

where y represents the dose at the coordinate x, whereas $x_{1}$ and $x_{2}$ represent the positions located at one‐third and two‐thirds of the half of FWHM from the central axis, respectively.

The parameters of CC13/PTW31010 and all other detectors were compared using Wilcoxon signed‐rank test using Microsoft Excel. Statistical significance was set at a *P* < 0.05.

## 3 RESULTS

**Figure 1:**
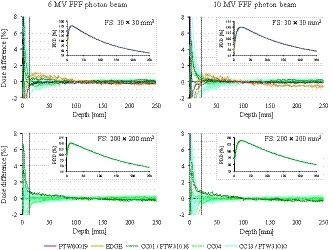
Percent depth dose curves of 6 and 10 MV flattening filter‐free (FFF) beams in the insets, as well as the dose differences between each curve and the mean values for the 30 × 30 and 200 × 200 mm^2^ field sizes. Dashed vertical lines in the dose difference plots show mean $d_{\max}$ (13 and 22 mm for 6 and 10 MV FFF beam) among all collected data

Figure 1 demonstrates the PDD curves measured using FSs of 30 × 30 and 200 × 200 mm^2^. The measured data are displayed in the insets, and the data of each institution subtracted the mean value of all the data are plotted in the main figure. In the dose fall‐off region, the variations were within ±1%, with the only exception being the region around the peak. Table 3 summarizes the SD_max_ values of the differences from the mean values. For all FSs, the SD_max_ values were within 0.65%. For regions deeper than 50 mm, SD_max_ was smaller than 0.4%.

**Table 3:** SD_max_ of the dose fall‐off region of the PDD

Energy	SD_max_ (mm^2^)		
	30 × 30	100 × 100	200 × 200
6 MV FFF beam	0.65% [N = 19]	0.48% [N = 20]	0.51% [N = 19]
10 MV FFF beam	0.63% [N = 18]	0.39% [N = 19]	0.40% [N = 18]

Abbreviations: FFF, flattening filter‐free; SD_max_, maximum standard deviation.

**Figure 2:**
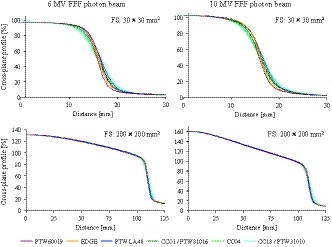
Off‐center ratio profiles of 6 and 10 MV flattening filter‐free beams measured at $d_{10}$ for the 30 × 30 and 200 × 200 mm^2^ field sizes

**Figure 3:**
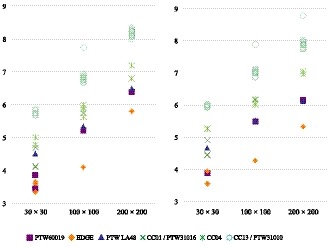
Penumbra width values of off‐center ratio profiles of 6 and 10 MV flattening filter‐free beams at $d_{10}$ measured with each detector for all field sizes. The penumbra widths are presented as the means of the right and left penumbra width values

Figure 2 illustrates the OCR profiles measured at $d_{10}$ with FSs of 30 × 30 and 200 × 200 mm^2^. Figure 3, meanwhile, depicts the mean penumbra width of the right and left side of the profile measured at $d_{10}$. The penumbra widths measured with the EDGE detector (Sun Nuclear Corp., Melbourne, FL, USA) exhibited approximately 30% decreased penumbra values compared to those of the CC13/PTW31010 detectors. The microDiamond detector (model 60019; PTW Freiburg) also demonstrated smaller penumbra widths for the 30 × 30 mm^2^ field. The penumbra width values of the OCR profiles measured with the CC13/PTW31010 detectors and all other detectors are summarized in Table 4. The penumbra widths are presented as the means of the right and left penumbra width values. Although the SD of the data measured with the CC13/PTW31010 detectors was <0.3 mm, those measured with all other detectors exceeded 0.6 mm. For all energies and FSs, the penumbra widths of the CC13/PTW31010 detectors were significantly larger than those of all other detectors (*P* < 0.05). Similar results were obtained for $d_{\max}$. Tables 5 and 6 summarize the analyzed values of the FWHM, unflatness, and slope calculated for OCR profiles measured with the CC13/PTW31010 detectors and all other detectors. Very small differences were observed between the values for the CC13/PTW31010 detectors and all other detectors. Similar results were found for $d_{\max}$. Although the slope of 6 MV FFF beams and the unflatness of 10 MV FFF beams with 200 × 200 mm^2^ FS showed statistically significant differences (*P* < 0.05), the differences were within 2%.

**Table 4:** Penumbra width values analyzed for OCR profiles of 6 and 10 MV FFF beams measured at $d_{10}$. Values analyzed for profiles measured with ionization chambers, including CC13 and PTW31010, and all other detectors are listed

Detectors	FS (mm)	6 MV FFF beam			10 MV FFF beam		
		30 × 30*	100 × 100*	200 × 200*	30 × 30*	100 × 100*	200 × 200*
CC13/PTW31010	Mean ± SD (min–max)	5.76 ± 0.08 (5.67–5.88) [N = 6]	6.87 ± 0.28 (6.67–7.73) [N = 12]	8.24 ± 0.25 (8.00–9.05) [N = 14]	5.97 ± 0.04 (5.92–6.03) [N = 6]	7.08 ± 0.26 (6.85–7.88) [N = 12]	7.95 ± 0.26 (7.74–8.78) [N = 13]
All other detectors	Mean ± SD (min–max)	4.10 ± 0.58 (3.34–5.01) [N = 11]	5.47 ± 0.62 (4.09–6.00) [N = 8]	6.54 ± 0.51 (5.80–7.18) [N = 5]	4.39 ± 0.65 (3.54–5.26) [N = 10]	5.67 ± 0.69 (4.26–6.19) [N = 7]	6.32 ± 0.70 (5.33–7.03) [N = 5]

Abbreviations: $d_{10}$, dose at a 10 cm depth; FS, field size; FFF, flattening filter‐free; min–max, minimum–maximum; OCR, off‐center ratio; SD, standard deviation.

* *P < *0.05.

**Table 5:** Parameters analyzed for OCR profiles of 6 MV FFF beams measured at $d_{10}$. Values analyzed for profiles measured with ionization chambers, including CC13 and PTW31010, and all other detectors are listed

Detectors	Parameter	FWHM (mm)		Unflatness (mm)		Slope	
	FS (mm)	100 × 100	200 × 200	100 × 100	200 × 200	100 × 100*	200 × 200*
CC13/PTW31010	Mean ± SD (min–max)	110.3 ± 0.4 (109.7–110.9) [N = 12]	220.8 ± 0.6 (219.9–221.6) [N = 14]	1.101 ± 0.002 (1.098–1.104) [N = 12]	1.228 ± 0.002 (1.224–1.231) [N = 14]	0.313 ± 0.003 (0.308–0.318) [N = 12]	0.377 ± 0.003 (0.372–0.385) [N = 14]
All other detectors	Mean ± SD (min–max)	110.0 ± 0.7 (109.1–111.0) [N = 8]	220.3 ± 0.6 (219.7–221.3) [N = 5]	1.102 ± 0.002 (1.100–1.106) [N = 8]	1.231 ± 0.004 (1.227–1.236) [N = 5]	0.318 ± 0.008 (0.305–0.328) [N = 8]	0.384 ± 0.009 (0.378–0.400) [N = 5]

Abbreviations: $d_{10}$, dose at a 10 cm depth; FFF, flattening filter‐free; FS, field size; FWHM, full width at half maximum; OCR, off‐center ratio; SD, standard deviation; min–max, minimum–maximum.

* *P *< 0.05.

**Table 6:** Parameters analyzed for OCR profiles of 10 MV FFF beams measured at $d_{10}$. Values analyzed for profiles measured with ionization chambers, including CC13 and PTW31010, and all other detectors are listed

Detectors	Parameter	FWHM (mm)		Unflatness (mm)		Slope	
	FS (mm^2^)	100 × 100	200 × 200	100 × 100	200 × 200*	100 × 100	200 × 200
CC13/PTW31010	Mean ± SD (min–max)	110.1 ± 0.4 (109.5–110.8) [N = 12]	220.5 ± 0.6 (219.6–221.3) [N = 13]	1.173 ± 0.002 (1.171–1.176) [N = 12]	1.419 ± 0.002 (1.416–1.425) [N = 13]	0.558 ± 0.006 (0.548–0.571) [N = 12]	0.705 ± 0.003 (0.699–0.709) [N = 13]
All other detectors	Mean ± SD (min–max)	109.8 ± 0.6 (109.0–110.9) [N = 7]	220.1 ± 0.6 (219.6–221.1) [N = 5]	1.173 ± 0.003 (1.168–1.175) [N = 7]	1.424 ± 0.005 (1.418–1.433) [N = 5]	0.561 ± 0.008 (0.550–0.571) [N = 7]	0.711 ± 0.010 (0.699–0.726) [N = 5]

Abbreviations: $d_{10}$,dose at a 10 cm depth; FFF, flattening filter‐free; FS, field size; FWHM, full width at half maximum; min–max, minimum–maximum; OCR, off‐center ratio; SD, standard deviation.

* *P *< 0.05.

**Figure 4:**
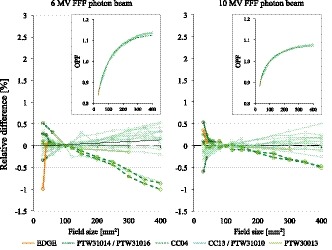
Output factor (OPF) curves of 6 and 10 MV flattening filter‐free beams are shown in the insets, with the relative differences between each curve and the mean values with field sizes ranging from 30 × 30 to 400 × 400 mm^2^

Figure 4 displays the OPF curves. The collected values are plotted in the insets, whereas the relative differences of each data point from the mean values are plotted in the main figure. The OPF curves measured with the different machines and detectors were consistent, and the relative differences were almost within 1% for all FSs, although the CC04 detector demonstrated the variations slightly exceeding 1% for the 6 MV FFF beam with ≥300 × 300 mm^2^ FSs.

## 4 DISCUSSION

In this study, we investigated the interunit variability of TrueBeam™ FFF beam data collected from multiple institutions, focusing on the FFF beam‐specific parameters. Glide‐Hurst et al. and Beyer et al. compared five and three TrueBeam™ linacs and reported that the small variabilities in PDD at a 10 cm depth were <0.3% and <1.0%, respectively.^21^, ^22^ Chang et al. also showed that the mean SD of the profiles derived from three TrueBeam™ machines was 0.4%.^17^ Tanaka et al. evaluated 21 TrueBeam™ machines’ beam data and reported that the difference between the mean beam data and RBD provided by the vendor was very small.^9^ Because the authors’ goal was to compare their data with the RBD, which was collected using a CC13 ionization chamber, they investigated only the data measured with the CC13 or PTW31010 semiflex ionization chambers. It is important to note that the data measured with the CC13 in the present study were also included in the study reported by Tanaka et al. The RBD are the mean beam data of three TrueBeam™ units collected at one institution. Although the RBD have been clinically used worldwide and can be used as a good reference data, we used the mean data generated from multiple institutions as a reference in this study. Although the data collected from multiple institutions include the interoperator variations, mean of multiple data will also provide an appropriate reference data. This study focuses on the detectors used for measurements affecting FFF‐specific parameters. To our knowledge, no study has thus far reported the interunit variability in FFF‐specific parameters.

In this study, we collected data measured with all detectors, including nonionization chamber detectors such as diode and diamond detectors, although most institutions used ionization chambers as the Eclipse TPS did not require small‐field data for modeling. Interunit variations come from the combined effects of linac unit, operator, and detector‐related causes. As shown in Figures 2, 3 and Table 4, the type of detector greatly affects the penumbrae of the OCR profiles. The CC13/PTW31010 ionization chambers showed significantly larger penumbra width than other detectors including diode and diamond detectors. The inner diameters of cavity of the CC13 and PTW31010 chambers are 5.5 and 6 mm, respectively, and they are much larger than the sensitive area of diode and diamond detectors (Table S1). Such differences of the sensitive volume greatly affected the measured data at steep regions. Similar data have previously been reported in many studies.^10^, ^11^ In contrast, the type of detector showed modest impacts on the shape of the curves, such as the dose fall‐off region of PDD and field region of OCR defined as 80% FWHM, probably because the dose variations are not steep. In addition, the FFF‐specific parameters, including unflatness and slope, showed very small variations among detectors. Although a few data showed statistically significant differences, the variations were within 2%. For flattened beams, the photon energy spectrum changes in off‐center region because of the thickness of flattening filter, whereas the spectrum of the FFF beams is not changed.^23^ Therefore, the energy spectrum will be stable in the field region where the FFF‐specific parameters are evaluated. However, it has been reported that large FS results in the increase of scattered photons with low energy, leading to an overresponse of diode detectors.^10^, ^24^ Our results showed that the interunit variations of the FFF beam profiles were small and remained unaffected by the selection of detectors; the only exception is the penumbra region of the OCR profile data. For middle–large FSs, however, CC13/PTW31010 ionization chambers will be still suitable because of the stable response against photon energies. The impact of variations in penumbra width is relatively small for large FSs.

The OPF also presented small variations of <1%. Although the data collected with CC04 showed smaller OPF values with larger FSs, almost all other data were within 0.5%. For small‐field dosimetry, many studies have demonstrated that the type of detector greatly affects the measured data. Alfonso et al.^25^ proposed output correction factors, which correct the effects of the detector type on small‐field dosimetry, and the International Atomic Energy Agency updated the factors in the Technical Reports Series no. 483.^12^ Akino et al. previously collected the beam data of Novalis Tx™ and reported that the OPF values varied in a detector‐dependent manner.^14^ In addition, the authors also showed that variations were significantly reduced when applying output correction factors, which indicates that the observed interunit variations primarily depended on the detectors used for the measurements. Although this study does not evaluate the effects of the output correction factors, the variations were almost within 1%. This is because we only evaluated FSs ≥ 30 mm as the Eclipse TPS did not require data for smaller FSs. For FSs ≥ 30 mm, corrections of OPF values are unnecessary.

## 5 CONCLUSIONS

In this study, we investigated the interunit variability in TrueBeam™ linacs among multiple institutions, focusing on FFF‐specific parameters and detector selection. Although the penumbra region demonstrated detector‐dependent variations, all other parameters, including the slope and unflatness, exhibited very small interunit variations, regardless of the detector type.

## CONFLICT OF INTEREST

The second author, Y. Akino, is a developer of the commercial software Akilles RT, which was used for analysis in this study.

## Supporting information


**Table S1**. Detector's characteristics.

## References

[1] Yamamoto M , Serizawa T , Shuto T , et al. Stereotactic radiosurgery for patients with multiple brain metastases (JLGK0901): a multi‐institutional prospective observational study. Lancet Oncol. 2014;15:387–395. 24621620 10.1016/S1470-2045(14)70061-0

[2] Chi A , Liao Z , Nguyen NP , Xu J , Stea B , Komaki R . Systemic review of the patterns of failure following stereotactic body radiation therapy in early‐stage non‐small‐cell lung cancer: clinical implications. Radiother Oncol. 2010;94:1–11. 20074823 10.1016/j.radonc.2009.12.008

[3] Boike TP , Lotan Y , Cho LC , et al. Phase I dose‐escalation study of stereotactic body radiation therapy for low‐ and intermediate‐risk prostate cancer. J Clin Oncol. 2011;29:2020–2026. 21464418 10.1200/JCO.2010.31.4377 PMC3138546

[4] Cashmore J . The characterization of unflattened photon beams from a 6 MV linear accelerator. Phys Med Biol. 2008;53:1933–1946. 18364548 10.1088/0031-9155/53/7/009

[5] Kragl G , af Wetterstedt S , Knäusl B , et al. Dosimetric characteristics of 6 and 10MV unflattened photon beams. Radiother Oncol. 2009;93:141–146. 19592123 10.1016/j.radonc.2009.06.008

[6] Dzierma Y , Licht N , Nuesken F , Ruebe C . Beam properties and stability of a flattening‐filter free 7 MV beam‐an overview. Med Phys. 2012;39:2595–2602. 22559630 10.1118/1.3703835

[7] Cho SH , Vassiliev ON , Lee S , Liu HH , Ibbott GS , Mohan R . Reference photon dosimetry data and reference phase space data for the 6 MV photon beam from varian clinac 2100 series linear accelerators. Med Phys. 2005;32:137–148. 15719964 10.1118/1.1829172

[8] TrueBeam Representative Beam Data for Eclipse . Varian Medical Systems. myvarian.com (last accessed, 8/22/2019).

[9] Tanaka Y , Mizuno H , Akino Y , Isono M , Masai N , Yamamoto T . Do the representative beam data for TrueBeamTM linear accelerators represent average data? J Appl Clin Med Phys/Am Coll Med Phys. 2019;20:51–62. 10.1002/acm2.12518 PMC6370991 30636358

[10] Akino Y , Gautam A , Coutinho L , Wurfel J , Das IJ . Characterization of a new commercial single crystal diamond detector for photon‐ and proton‐beam dosimetry. J Radiat Res. 2015;56:912–918. 26268483 10.1093/jrr/rrv044 PMC4628217

[11] Marsolat F , Tromson D , Tranchant N , et al. A new single crystal diamond dosimeter for small beam: comparison with different commercial active detectors. Phys Med Biol. 2013;58:7647–7660. 24113353 10.1088/0031-9155/58/21/7647

[12] IAEA TRS‐483 . Dosimetry of small static fields used in external beam radiotherapy: an IAEA‐AAPM International Code of Practice for reference and relative dose determination, Technical Report Series No. 483. Vienna, Austria: International Atomic Energy Agency; 2017. 10.1002/mp.13208 30247757

[13] ICRU . Prescribing, recording, and reporting of stereotactic treatments with small photon beams (ICRU Report 91). Bethesda, MD, U.S.A.: International Commission on Radiation Units and Measurement Report No. 91; 2017.

[14] Akino Y , Mizuno H , Tanaka Y , Isono M , Masai N , Yamamoto T . Inter‐institutional variability of small‐field‐dosimetry beams among HD120TM multileaf collimators: a multi‐institutional analysis. Phys Med Biol. 2018;63:205018. 30255847 10.1088/1361-6560/aae450

[15] Dalaryd M , Knoos T , Ceberg C . Combining tissue‐phantom ratios to provide a beam‐quality specifier for flattening filter free photon beams. Med Phys. 2014;41:111716. 25370630 10.1118/1.4898325

[16] Akino Y , Ota S , Inoue S , et al. Characteristics of flattening filter free beams at low monitor unit settings. Med Phys. 2013;40:112101. 24320454 10.1118/1.4824920

[17] Chang Z , Wu Q , Adamson J , et al. Commissioning and dosimetric characteristics of TrueBeam system: composite data of three TrueBeam machines. Med Phys. 2012;39:6981–7018. 23127092 10.1118/1.4762682

[18] Lang S , Hrbacek J , Leong A , Klock S . Ion‐recombination correction for different ionization chambers in high dose rate flattening‐filter‐free photon beams. Phys Med Biol. 2012;57:2819–2827. 22510780 10.1088/0031-9155/57/9/2819

[19] Kry SF , Popple R , Molineu A , Followill DS . Ion recombination correction factors (P(ion)) for Varian TrueBeam high‐dose‐rate therapy beams. J Appl Clin Med Phys/Am Coll Med Phys. 2012;13:318–325. 10.1120/jacmp.v13i6.3803 PMC5718527 23149774

[20] Fogliata A , Garcia R , Knoos T , et al. Definition of parameters for quality assurance of flattening filter free (FFF) photon beams in radiation therapy. Med Phys. 2012;39:6455–6464. 23039680 10.1118/1.4754799

[21] Glide‐Hurst C , Bellon M , Foster R , et al. Commissioning of the Varian TrueBeam linear accelerator: a multi‐institutional study. Med Phys. 2013;40:031719. 23464314 10.1118/1.4790563

[22] Beyer GP . Commissioning measurements for photon beam data on three TrueBeam linear accelerators, and comparison with Trilogy and Clinac 2100 linear accelerators. J Appl Clin Med Phys/Am Coll Med Phys. 2013;14:273–288. 10.1120/jacmp.v14i1.4077 PMC5714054 23318395

[23] Dalaryd M , Kragl G , Ceberg C , et al. A Monte Carlo study of a flattening filter‐free linear accelerator verified with measurements. Phys Med Biol. 2010;55:7333–7344. 21081829 10.1088/0031-9155/55/23/010

[24] Yin Z , Hugtenburg RP , Beddoe AH . Response corrections for solid‐state detectors in megavoltage photon dosimetry. Phys Med Biol. 2004;49:3691–3702. 15446798 10.1088/0031-9155/49/16/015

[25] Alfonso R , Andreo P , Capote R , et al. A new formalism for reference dosimetry of small and nonstandard fields. Med Phys. 2008;35:5179–5186. 19070252 10.1118/1.3005481

