# Supplementary material for: Impact of detector selections on inter‐institutional variability of flattening filter‐free beam data for TrueBeam™ linear accelerators
Source: J Appl Clin Med Phys. 2019 Nov 18;21(1):36–42. doi: 10.1002/acm2.12766 (PMC6964765; doi:10.1002/acm2.12766)
Supplement: Supplementary file 1 — Table S1. Detector's characteristics. [file ACM2-21-36-s001.pdf]

Supplementary Table 1: Detector's characteristics

Detector	microDiamond (model 60019)	EDGE (model 1118)	LA48	CC01	PinPoint (model 31014)	PinPoint 3D (model 31016)	CC04	Semiflex (model 31010)	CC13	Farmer (model 30013)
Vendor	PTW	Sun Nuclear	PTW	IBA	PTW	PTW	IBA	PTW	IBA	PTW
Type	Diamond	Shielded diode	Linear chamber array	IC	IC	IC	IC	IC	IC	IC
Sensitive volume	–	–	–	0.01 cc	0.015 cc	0.016 cc	0.04 cc	0.125 cc	0.13 cc	0.6 cc
Sensitive area/Inner diameter	2.2 mmϕ, disk	0.8 × 0.8 mm	4 × 4 mm	2 mm	2.9 mm	2.9 mm	4 mm	5.5 mm	6 mm	6.1 mm

Abbreviation: IC, ionization chamber
